# Parental Stress and Family Quality of Life: Surveying Family Members of Persons with Intellectual Disabilities

**DOI:** 10.3390/ijerph17239007

**Published:** 2020-12-03

**Authors:** Cristina Jenaro, Noelia Flores, Belén Gutiérrez-Bermejo, Vanessa Vega, Carmen Pérez, Maribel Cruz

**Affiliations:** 1 INICO/Faculty of Psychology, Universidad de Salamanca, 37005 Salamanca, Spain; nrobaina@usal.es; 2 Faculty of Psychology, Universidad Nacional de Educación a Distancia (UNED), 28040 Madrid, Spain; mbgutierrez@psi.uned.es; 3 Faculty of Education, Pontificia Universidad Católica de Valparaíso, Viña del Mar 1290, Chile; vanessa.vega@pucv.cl; 4 Faculty of Nursing, Universidad Autónoma de San Luis Potosí, San Luis Potosí 78240, Mexico; ma.perez@uaslp.mx (C.P.); maribel.cruz@uaslp.mx (M.C.)

**Keywords:** intellectual disability, Parenting Stress Index–Short Form, parental stress, family quality of life, Beach Center Family Quality of Life Scale

## Abstract

(1) Background. This study assesses the quality of life in families with a member with an intellectual disability using the Family Adjustment and Adaptation Response framework. (2) Methods. The study included 515 Spanish participants whose family members with disabilities range in age from infancy to adulthood. We hypothesized that it is possible to predict parenting stress by paying attention to the meaning families give to themselves and their circumstances while controlling for the impact of other variables such as family capabilities and characteristics of the family member with disabilities. We used the Beach Center Family Quality of Life Scale and the section on Exceptional needs of medical and behavioral support from the Supports Intensity Scale, together with other potential predictors. The subscale on parental stress from the Parenting Stress Index–Short Form was utilized as a criterion measure. (3) Results. Hierarchical multiple regression analysis revealed that 49% of parental stress was predicted by dysfunctional interaction, difficult behaviors, low emotional wellbeing, poor family interaction, as well as kinship as parents, and the severity of both the medical needs and intellectual disability. (4) Conclusions. The stress experienced by those families is mostly predicted by the meaning they give to themselves and their circumstances. Implications of these findings for service delivery are discussed.

## 1. Introduction

Caring for a family member with a disability, as well as responding to and supporting his/her transition to adulthood needs, involves many challenges. Not surprisingly, a significant number of studies suggest that these families experience more stress [1,2,3]. The prominent role of the parents as primary caregivers of their children with disabilities has allowed the development of numerous studies focused on their experiences [2,4,5,6]. In turn, the development of frameworks, such as Family Stress and Family Quality of Life (FQoL), has provided empirical evidence on the impact of different features on family dynamics and the resulting adjustment or mismatch. Of special interest is to identify which characteristics help these families cope with the stressors associated with the additional needs and supports that family members with disabilities will require over time. 

Here, the Family Adjustment and Adaptation Response (FAAR) Model [7,8,9] allows us to analyze the features associated with achieving family adjustment or adaptation. As Patterson summarizes [7], according to the FAAR Model, families engage in active processes to balance demands with capabilities. These interact with family meanings to reach a certain level of family adjustment or adaptation. Four central constructs are relevant in the FAAR Model: (1) family demands, (2) family capabilities, (3) family meanings, and (4) family adjustment or adaptation. Family demands are comprised of normative and non-normative stressors, strains, and daily hassles. Family capabilities include tangible and psychosocial resources and coping behaviors. In turn, family meanings relate to their appraisal of the demands, of their cohesion, and of themselves in relation to external systems. Finally, family adjustment or adaptation reflects the outcome of this active process to balance family demands with family capabilities as they interact with family meaning [7]. Following Patterson [7], the process by which families face crisis and restore balance is called regenerative power in stress theory, and family bonadaptation in the FAAR and in the Family Resilience Model [10]. To measure family adjustment vs. stress, one of the most utilized measures is the Parenting Stress Index–Short Form (hereafter referred to as PSI/SF) [11], which assesses parents’ factors, such as their sense of competence, characteristics of their child, such as their temperament, needs, and adaptability, and their interactions. The PSI/SF does not measure external factors such as supports. This shortcoming is overcome when the FQoL concept is included.

Accordingly, results from a scoping review [12] identified five factors that affect FQoL, namely, (a) disability-related support, (b) family interactions/family relationships, (c) overall well-being, (d) support from services, and (e) severity and type of disability. The review also identified that one of the most frequently used scales for measuring FQoL within services for children was the Beach Center Family Quality of Life Survey [13] (hereafter referred to as BC-FQOL Scale). This measure assesses family functioning in five domains: Family interaction, Parenting, Emotional well-being, Physical/material well-being, and Disability-related supports [14]. The first domain, Family interaction, assesses family cohesion. The second domain, Parenting, refers to the support provided by family members to the children with disabilities (e.g., helping them to be independent); it relates to social support. The third domain, Emotional well-being, reflects feelings of satisfaction and support. The fourth domain, Physical/material well-being, refers to family access to education, health, social services, and the like. The fifth domain, Disability-related support, reflects the family appraisal of having formal (e.g., having good relationships with the service providers) and informal supports (e.g., having the support to accomplish goals at home) for the member with disabilities. 

The focus of the FQoL construct on the family as a unit prevents the BC-FQOL Scale from informing on the severity and type of disability of the family member. As we mentioned earlier, severity and type of disability is a well-known factor affecting FQoL, so to achieve a complete picture of the degree of family adaptation with a member with a disability, it is necessary to include this factor in the analysis. This is achieved through the concept of parenting stress, which has been studied for several decades. Analyzing the existing studies, most of them on children with an autism spectrum disorder, it is possible to identify several factors associated with stress in parents of children with intellectual/developmental disabilities. The first group of characteristics is related to the parents/family such as: (1) sociodemographic characteristics [15,16,17], and (2) skills and cognitive features such as coping and locus of control [1,2,18,19,20], sense of coherence [21], wellness, children attachment [22], and parenting styles [23]. The second group of features relates to the family member with intellectual/developmental disabilities. It comprises: (1) medical needs [24,25] and behavioral problems [15,26,27,28,29]. Finally, the third set of factors relates to external support (social, economic, etc.) [15,16,20].

Regarding parents’ characteristics, sociodemographic features, such as economic [15,16], education, and employment status, affect the experience and response to these demanding situations [17,30,31]. Age has also been shown to impact the stress experienced by family members, and the older the parents the higher the support needs [30,32]. In addition, families with children with intellectual disabilities whose parents have full-time jobs, higher income, and educational level experience higher FQoL [33,34,35].

With regards to the family member with disabilities, some features, such as the type and severity of the disability, and associated behavioral and health issues have been highlighted in numerous studies, and the more severe and complex the issues, the higher the family stress [6,15,18,26,27,28,29,36,37,38]. The severity of the intellectual disability has been found associated with the caregiver’s FQoL [6,34]. Concerning age, in a study carried out in Spain, it was found that FQoL was higher in families with young adults with intellectual disabilities than in families with comparable children up to 18 years of age [33]. In another study with young adults with intellectual and/or developmental disabilities, data show that lower frequency of challenging behaviors and lower support needs were associated with higher FQoL [39].

External and intra-family supports are powerful features in favoring a more or less adaptive response to the demands of caring for relatives with disabilities [40,41]. Positive perceptions toward the situation and the family member with intellectual disability reduce parenting stress (i.e., inverse and significant associations) [1,2,18,19,20] and raise levels of FQoL (i.e., direct and significant associations) [42]. Emotional support from family members has been found associated with higher FQoL [43,44]. Similarly, processes inside the family related to family interaction or cohesion are predictors of stress in parenting a child with a disability [17,45,46,47]. This issue has been used to explain differences in wellbeing in families of children with disabilities [46,47,48,49]. Concerning external supports, scientific literature offers contradictory results. While some studies suggest that formal supports do not significantly impact parents’ ratings of confidence in parenting [40], other studies suggest that access to services promotes family adjustment [31,45,50,51] and that combined formal and informal support leads to greater happiness [52], whereas the lack of opportunities for social inclusion for adults with intellectual disabilities has been found associated to lower FQoL [53]. Moreover, social support reduces parent stress [15,16], as does access to services [20].

**Figure 1:**
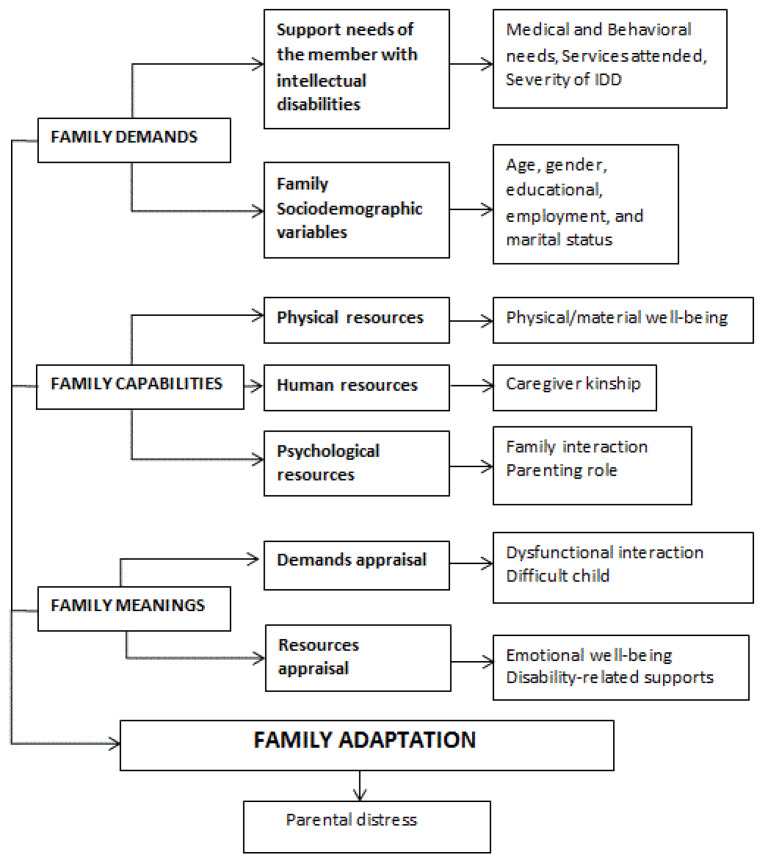
Relevant variables in the process of family adaptation to the care of a relative with a disability. IDD = Intellectual and Developmental Disabilities.

Although family stress and FQoL in family members with intellectual and/or developmental disabilities have been studied separately, there is a scarcity of studies combining both frameworks. The existing studies are focused on children [37,54,55] and there is a lack of research on a broader age range. As a step forward, in this article, we try to integrate the multidimensional view of the FQoL construct, as measured by the BC-FQOL Scale, with the FAAR model, as measured by the PSI/SF scale, to predict family adaptation, as reflected in Figure 1. Note that the key domains of the FAAR model appear in capital letters. The concepts included in each domain are indicated in bold and lowercase. The normal font is used to indicate potentially relevant variables selected for the current study.

According to the FAAR model, everything concerning family members with a disability, from the diagnosis to their different medical and behavioral needs, can be considered a non-normative stressor. Its impact is modulated by the family’s capabilities, that is, what the family has (resources) and what the family does (behaviors) [7]. It is also modulated by how families perceive themselves and their circumstances (appraisal). Depending on the interplay between all these different features, the end result of the process can lead to different levels of family adjustment–maladjustment.

Taking into account the above, in this study, we aim at analyzing the degree to which the demands, resources, and meanings that families give themselves and their circumstances contribute to predict family adaptation. More specifically, we hypothesize that:

(1) Family demands will contribute significantly to predict family adaptation, and there will be a negative and significant association between those variables.
(2) Family capabilities, controlled for family demands, will contribute significantly to predicting family adaptation, and there will be a positive and significant association between those variables.
(3) Family meanings, after controlling for demands and resources, will contribute significantly to the prediction of family adaptation, and the more positive the evaluation, the higher the family adaptation.

In short, and as we will explain in more detail next, we are combining two approaches (i.e., FAAR and FQoL) and utilizing three measures well-known from their respective fields to help better understand how families try to find a balance between demands and resources in their quest to provide supports that meet the needs of their family members with intellectual disabilities. To our knowledge, although there is some research about this [55,56], there are no published articles that have used such a combination of measures. Nor are we aware of studies that have incorporated these measures into a theoretical framework such as the FAAR.

## 2. Materials and Methods

### 2.1. Procedure

Data were gathered from 2015 to 2016. The study required the approval and support from Plena Inclusión, the Spanish National Confederation of Organizations for Persons with Intellectual and Developmental Disabilities. Plena Inclusión is an organization that represents people with intellectual or developmental disabilities and their families in Spain. Each region has its federation of associations; in total there are almost 900 associations in Spain. This organization provides support to 140,000 people with intellectual and/or developmental disabilities and their 235,000 family members. The organization has a staff of 4000 professionals. Their support to carry out the study required signing a cooperation agreement. Plena Inclusión informed all its partner organizations. Those that agreed to participate were by the Confederation. The evaluation dossier was sent by Plena Inclusión by post to all families of the associations interested in participating. A total of 580 questionnaires were distributed among 25 organizations, in eight different autonomous communities or regions. Of these, 519 questionnaires (89.5%) were returned to the research team in closed envelopes to ensure the privacy of the data. The good relationship with the families, the anonymity of the responses, the interest in the topic, and the ease of the response procedure and delivery of reports explain the high participation and response rate. After confidentiality and anonymity were guaranteed, from a total of 519 returned questionnaires sent, 4 questionnaires (0.8%) were excluded from the current analyses because the informants did not provide complete sociodemographic information or they did not respond to all items of the included measures. For participating in the study, each organization received a report with comprehensive data and information relating to its center, so as to help them implement strategies to enhance the quality of family life.

### 2.2. Participants

Data from a total of 515 relatives of individuals with intellectual disabilities were analyzed in the current study. The following inclusion criteria were established: (1) acceptance of the association to participate in the study, (2) acceptance of families to respond to the evaluation dossier by returning it by post, (3) informed consent of the families (i.e., each informant signed the consent form attached to the assessment dossier when returning it), (4) being a relative of a currently living person with intellectual and/or developmental disabilities, (5) the family member with disabilities had to be residing with the family or in a community alternative, that is, they should not be institutionalized. Non-compliance with any of these criteria established exclusion. Under the term “intellectual disability”, different conditions, as classified according to the ICD-11 [57], are termed as Disorders of intellectual development (6A00). Disorders of intellectual development are a group of etiologically diverse conditions originating during the developmental period characterized by significantly below average intellectual functioning and adaptive behavior. In the DSM-5 manual [58], the term Intellectual Disability (319, F79) is used. The following requirements must be met for its diagnosis: Deficits in intellectual functions and in adaptive functioning, as well as the onset of deficits during the developmental period. Finally, The American Association on Intellectual and Developmental Disabilities (AAIDD) [59], a world-leading organization in the field, defines intellectual disability as a disability characterized by significant limitations in both intellectual functioning and in adaptive behavior, which covers many everyday social and practical skills. This disability originates before the age of 18. The AAIDD also states that the term ‘developmental disabilities’ is a broader category of an often lifelong disability that can be intellectual, physical, or both.

Families were advised to choose the person most knowledgeable about the person with intellectual disabilities as the respondent, as only one survey was gathered for each family. Ages of informants ranged from 20 to 90 (Mean = 54.1; SD = 12.1). As Table 1 summarizes, 73.6% were parents of individuals with intellectual disabilities, 19.8% were brothers or sisters, and 6.8% have other relationships (e.g., cousin, aunt…). The ages of the individuals with disabilities ranged from 4 to 72 years (Mean = 30.5; SD = 13.3), with a medium intensity of behavioral support needs of 14.5 (SD = 2.6; range: 13 to 35), and a medium-low intensity of medical support needs of 11.2 (SD = 1.9; range: 10 to 30). All lived with their families. Information on the severity of the intellectual disability was provided by the informants as this data can be obtained from medical records. Worldwide, four levels of severity are distinguished: (1) Mild, for individuals with IQ (i.e., intelligence quotient) from 50–55 to 70, (2) Moderate, when IQ ranges from 35–40 to 50–55, (3) Severe, for IQ between 20–25 to 35–40, and (4) Profound, for IQ less than 20 or 25. As Table 1 shows, almost 46% of the family members with intellectual and/or developmental disabilities have moderate levels of severity. Informants were requested to report the number of services attended by their relatives with intellectual and/or developmental disabilities. These services were grouped into: (1) daycare centers, (2) training/educational centers, (3) employment, (4) leisure and free time services. As can be seen in Table 1, almost 52% of relatives attended/utilized one type of service only.

### 2.3. Measures

(1) Sociodemographic questionnaire. The first section of the assessment dossier included several questions on the respondent and the relative with intellectual disabilities, such as gender, age, and educational status. It also allowed us to gather information on the severity of the intellectual disability (i.e., mild, moderate, severe, and profound) as registered in their personal records [60]. All those variables are part of the dimension of Family demands. In addition, caregiver kinship is considered a human resource within the dimension of Family capabilities in our theoretical model (see Figure 1).

(2) The PSI/SF [11] is a 36-item scale that assesses parental stress that has been utilized worldwide and is proven to be useful in families of children with different intellectual and/or developmental disabilities [2,26,27,28,29,56,61]. The Spanish version has shown adequate psychometric properties for assessing stress in children under 12 years [62,63], children aged 0 to 8 years [64], and the stress of relatives of individuals with intellectual disabilities aged from 4 to 72 years [65]. The items are grouped into three subscales whose reliability indexes for the current study are included in parenthesis: (1) Difficult child (α = 0.89) measures a child’s self-regulatory abilities as perceived by the parent; (2) Parent–child dysfunctional interaction (α = 0.78) assesses parental dissatisfaction of interactions with the child and the degree to which parents find it unacceptable. These two subscales are considered a Demands appraisal, belonging to Family meanings in our model (see Figure 1), in terms of behaviors (i.e., difficult child) and patterns of interaction (i.e., dysfunctional interaction). The third factor, (3) Parental distress (α = 0.88) [66], assesses levels of distress resulting from personal factors such as depression or conflicts with a partner and life restrictions due to the demands of child-rearing. It is related to feelings of loss of control, and dissatisfaction. This subscale is used as a criterion variable of family adaptation, with higher scores denoting higher stress/maladaptation (see Figure 1). Cronbach’s alpha in the current study for the total scale was 0.92.

(3) The BC-FQOL [13] was validated for Spanish families of children with intellectual disabilities, between 0 and 6 years old [67,68]. As explained earlier, this measure assesses family functioning with five domains. Although the measure asks to rate the importance and satisfaction with all the family domains, in the present study, we only utilized data on satisfaction, as we are identifying family adaptation/well-being rather than the perceived relevance of the different domains. As we were utilizing the measure with family members with intellectual disabilities of a broader range of ages, we first used confirmatory factor analysis to verify whether the data collected adjusted to the model. We proceeded to estimate the hypothesized model by using the DWLS estimation method, from the matrix of polychoric correlations. Table 2 summarizes the fit indexes for the measure. These data support the multidimensionality of the proposed model with the studied participants.

Once the factor structure of the scale was confirmed, the internal consistency of the subscales was tested. We obtained Cronbach’s alpha = 0.80 for Physical/material well-being; α = 0.87 for Family interaction, and α = 0.81 for Parenting role. In addition, we obtained α = 0.74 for Emotional well-being, and α = 0.80 for Disability-related support. Cronbach’s alpha for the total measure was 0.93. These data are comparable to those obtained in previous studies with the Spanish version [33,69] and support its reliability. In the current study, three subscales, namely, Family interaction, Parenting, and Physical/material well-being, were considered Resources/capabilities of the family, given that they relate to how well they get along, how well adults support children, and how many external resources they have. The remaining two scales, Emotional well-being and Disability-related supports, were included in the Family meaning category, as they relate to Resources appraisal and more specifically to feelings of balance/satisfaction with what they have as a family or with the support they receive for the member with a disability (see Figure 1).

(4) The section on Exceptional needs for medical and behavioral support from the Supports Intensity Scale—SIS [70,71], Spanish version [72]. This section lists 16 different medical conditions (e.g., respiratory care) and 13 problem behaviors (e.g., self-injury) commonly associated with intellectual disabilities to be rated 0 (no support required), 1 (some support required), or 2 (extensive support required). Consequently, higher scores denote higher support needs. Both factors were included in the domain of Family demands in our model (see Figure 1). As the authors of the scale state, an underlying assumption is that certain medical conditions and challenging behaviors predict that a person will require increased levels of support, regardless of her or his relative intensity of support needs in other life areas such as Home Living, Community Living, Life-long Learning, Employment, Health and Safety, and Social activities [67,68]. It is a support needs assessment scale and is not a scale to measure personal competence. It is assumed that a direct measure of support needs is more useful to determine how to best support an individual in community settings. Both variables are considered clinical variables of the family member with a disability that is part of the family demands.

### 2.4. Design and Analyses

First, test statistics, such as Cronbach’s alpha, and confirmatory factor analyses were utilized. Next, for this cross-sectional study, descriptive analysis, together with bivariate (Pearson’s Correlations) and multivariable analyses, was utilized as well. More specifically, to test our hypotheses, a multiple hierarchical regression analysis was done to assess the variables that, as displayed in Figure 1, could predict family adaptation in terms of stress. According to this method, the sets of variables or blocks will be added one at a time to determine if adding each of these blocks significantly improves the model’s ability to predict the criterion variable, that is, parental stress. The last block includes the variables of greatest interest in the present study, in terms of their predictive power of the criterion variable.

## 3. Results

First, selected variables were correlated to scores in the criterion variable, parental distress subscale of the Parenting Stress Index–Short Form (Table 3). As can be noted, most of the sociodemographic variables were not significantly associated with the criterion variable. Specifically, family sociodemographic characteristics did not correlate to parental stress. Similarly, general demographic information of the member with intellectual and/or developmental disabilities did not correlate to parental stress either. Since the associations in a bivariate analysis may change in a multivariate analysis, we have estimated the fully specified model to assess whether non-significant variables in the univariate analysis continue to be non-significant on the fully specified model (see Supplementary Table S1). Given the little explanatory capacity that this model adds with respect to the one we propose (*R*^2^ = 0.507 vs. *R*^2^ = 0.490), and following the principle of parsimony, we have maintained our proposed model. Next, the data were tested for multicollinearity. Additionally, the linearity of relations was assessed by visual inspection of scattergrams. The normality of the error distribution was assessed by a visual inspection of residuals through histogram and P-P normal graphic. The independence of errors was assessed with the Durbin-Watson test (DW = 2.01). Lastly, the homoscedasticity of the errors was assessed with a visual inspection of residuals. Outliers were also identified to check their possible effect on the regression.

The eleven independent variables that significantly correlated to Parenting distress, grouped into three blocks, were included in the analysis. The first block (support needs) included both Behavioral and Medical needs and Severity of intellectual disabilities. The second block (family capabilities) included Physical/Material well-being, Kinship with the member with intellectual disability, Family interaction, and Parenting. The third block (Family meaning) included two of the three subscales of the Parenting Stress Scale, namely, dysfunctional interaction and difficult behaviors, as well as two of the five subscales of the BC-FQOL: Emotional well-being, and Disability-Related Support. For the analysis, a total of 509 participants were considered as six outliers were identified. The total in Parental distress subscale was the criterion variable. Table 4 summarizes the regression.

Based on the semi-partial correlation squared *sr*^2^ values, the percentage of the total variance in the dependent variable uniquely accounted for by each independent variable at its point of entry is indicated in parenthesis. The first block (support needs) explained 6.4% of the total variance and behavioral needs (2.5%) as well as the severity of the intellectual disability (1.3%) had a significant effect. When the second block (family capabilities) is included, the determination coefficient reached 20.5%, indicating that Family capabilities, specifically kinship (2.6%), and family interaction (3.0%), controlling for support needs, explained 14.1% of the variance. When the third block was aggregated, the percentage of explained variance reached 49%, indicating that family appraisal, and specifically demands appraisal related to dysfunctional interaction (5.9%), and difficult behaviors of the relative with intellectual disabilities (3.5%), as well as resources appraisal related to emotional well-being (1.5%), explained 28.5% of the variance. The overall model accounted for 49% of the variance in parental stress and included as predictors of parental distress, in order of importance: dysfunctional interaction, difficult behaviors, low emotional wellbeing, poor family interaction, kinship as parents, high severity of medical needs, and high severity of intellectual disabilities.

The significance of the model was tested to contrast the null hypothesis “omnibus” by using the F test of the last block in the regression. The final model was significant (F(10,498) = 46.745; *p* < 0.0001). In summary, the family appraisal on resources and demands, together with their psychological and human resources as family, help predict distress vs. adaptation in families with individuals with disabilities. Though, as expected, after overlapping effects were removed, the severity of both medical needs and intellectual disabilities helped predict parental distress as well.

## 4. Discussion

The present study has tried to increase the existing knowledge about the features associated with parental stress in families with members with intellectual disabilities. It should be noted that unlike existing studies, this one has a large and diverse sample in terms of ages (from infancy to old age) with a great diversity in levels of intellectual disability (from mild to profound). It also includes participants from various regions of Spain, so the results better reflect this diversity.

The study has been carried out from a theoretical framework of parental stress, the FAAR model. In addition, it has been combined with the FQoL framework in order to more completely investigate the aspects associated with family adaptation when there is a family member with an intellectual disability. In contrast to the studies focused specifically on stress theories, which emphasize the role of coping strategies, we expand our focus to include more diverse features such as family perceptions, as well as family strengths. Likewise, compared to studies on FQoL that typically use a measure developed under the quality of life framework [73,74], this study integrates the dimensions of the BC-FQOL into the FAAR model. From this organizing framework, we expected that the demands or stressors related to the person with intellectual disabilities, in combination with the resources available to the family and with the interpretation or meaning that they give to their family and their circumstances, would contribute to predicting parental adaptation (more specifically, in this case, the stress in the performance of the parental role).

Indeed, the results indicate that the characteristics associated with the person with intellectual disability, such as the existence of associated medical problems and the presence of greater severity of the intellectual disability, contribute to predicting parental stress, even when other family factors are taken into account. The weight of medical issues has been found in previous studies with children with physical conditions [24,25,75]. The severity of intellectual disabilities has been found associated with FQoL [6,34] so that it can be interpreted as evidence of its association with family adjustment. The weight of these characteristics is, however, less than that of other features. This is important as it offers avenues to support families. Thus, enhancing the capabilities of families and helping them make sense of their circumstances is a clear way to improve their adaptation.

Focusing on family resources, the results suggest that family interaction patterns, followed by the relationship that exists with the person who assumes the main caregiver tasks, are also relevant aspects. Family interaction, as assessed by the BC-FQoL, is related to feelings of cohesion and mutual support. Cohesion is a family resource that has been identified in the literature on families of children with intellectual disabilities [76] and it is associated with families’ well-being [48] and FQoL [77]. Being parents, compared to being siblings or having another relationship with the person with intellectual disabilities, also deserves attention. Given that neither the age of the caregiver nor that of the family member with disability were significantly associated with parental stress, it is possible to hypothesize that it is rather the responsibility of being parents that increases stress. The roles associated with parenting such as protector, provider, defender, and representative place parents in a situation of special vulnerability to stress, which also requires additional support. Empowering parents and boosting their self-esteem is the key to achieving better family adjustment [78,79]. Although the characteristics discussed so far play a relevant role in the prediction of parental stress, the set of variables that explain a greater part of the variance of this stress are those related to the meaning that families give to their circumstances: first, the perception of having a dysfunctional interaction with the family member with a disability that relates to feelings of failure as a parent and failure to meet expectations on the part of the child; second, the perceptions of inappropriate behavior that causes discomfort in parents; and third, the perception that the family lacks sufficient support (friendship, respite, outside help). All these features are cognitive in nature and have been identified in previous studies associated with family stress and FQoL [18,47,76,80,81].

The identification of misaligned processes (e.g., in family patterns of interaction) can serve as a wake-up call for the implementation of services including family counseling or family therapy. For its part, the identification of vulnerabilities requires social, political, economic, etc., initiatives to palliate a disadvantaged situation. Moreover, the finding of an absence of formal or informal supports should be used to prioritize the provision of both general (e.g., education, health) and disability-related services (e.g., personal assistants, equipment, etc.). The expected outcomes of all these efforts should lead to an improvement in the objective conditions of the family, from the most basic (physical, psychological, and financial health) aspects to those related to the elimination of situations of social exclusion and lack of opportunities for growth and personal development.

Some final notes of caution should be emphasized. First, the sample selection procedure limits the generalizability of the results. Second, the diversity of the studied sample in terms of the demographic information, both from the informants and the individuals with intellectual disability (see Table 1), may question the extent to which the results obtained with the sample would be considered globally replicable if more homogeneous subgroups were analyzed. In the present study, we have chosen to respect the diversity of conditions that occur naturally in the centers and services that work with this population. Further studies, with ad hoc sample sizes large enough, will allow confirming or refuting these findings. Third, regarding the measures used, in the current study, we have utilized a limited number of measures. Future studies could include measures to specifically assess family characteristics such as resilience [82] coping strategies [47,83,84] and social skills [85]. Additional measures to assess cognitive features, such as a sense of coherence and self-esteem ([18,47,76,80,81], could help obtain a more comprehensive view of family adaptation and related features. Finally, the analyses carried out do not allow ruling out the possibility that the results are affected by endogeneity (explanatory variables correlated with the error terms), inverse causality (the criterion variable influencing the predictor), or simultaneity (both influence each other). This could lead to biased and inconsistent parameter estimates. Although the present study exposes the causal nature of relationships from a theoretical point of view, future studies with designs that allow investigating causality in relationships from an empirical point of view (e.g., longitudinal studies) will allow confirming or refuting the present findings [86].

Planning for supports and services should focus on individuals with disabilities and their families. Therefore, the assessment of all the aspects discussed here is essential for identifying family strengths and needs. Then, supports at the individual, family, and social level will not only reduce parenting stress but also will improve FQoL.

## 5. Conclusions

The present study expands the existing knowledge about parental stress by using a large and diverse sample of families and their relatives with intellectual disabilities. It offers a theoretical framework that allows combining the use of parental stress and family quality of life tools. More importantly, it identifies relevant variables to be considered when planning for supports. The study also highlights the importance for families to understand their circumstances and staying together to achieve better adaptation.

## Figures and Tables

**Table 1:** Demographics of the informants and individuals with disabilities.

Variables (and Codes)	*N*	%
Gender of caregivers		
Male (1)	158	30.7
Female (2)	357	69.3
Caregivers kinship		
Parent (1)	379	73.6
Sibling (2)	101	19.8
Other (3)	35	6.8
Marital status of caregivers		
Married (1)	364	70.7
Living alone (widow, single, divorced) (2)	117	29.0
Educational status of caregivers		
Primary education (1)	191	37.1
Secondary education (2)	144	28.0
High school (3)	58	11.3
University studies (4)	122	23.7
Employment status of caregivers		
Working (1)	211	41.0
Not Working (2)	237	59.0
Gender of individual with disability		
Male (1)	308	59.8
Female (2)	207	40.2
Severity of Intellectual disability		
Mild (1)	97	19.8
Moderate (2)	234	45.4
Severe (3)	159	30.9
Profound (4)	25	4.9
Number of services attended (occupational, recreational…)		
None (0)	10	1.9
One (1)	285	51.5
Two (2)	155	30.1
Three (3)	60	11.7
Four (4)	25	4.9

Note: to facilitate the interpretation of the results, the codification utilized for the different variables has been included in parenthesis.

**Table 2:** Goodness-of-fit statistics for the Five Factors Model of Family Stress and Family Quality of Life (FQOL) Scale.

Goodness-of-Fit Statistics	Values
Degrees of Freedom	256
Satorra-Bentler Scaled Chi-Square (*p* = 1.00)	35.21
Root Mean Square Error of Approximation (RMSEA)	0.0
P-Value for Test of Close Fit (RMSEA <0.05)	1.00
Expected Cross-Validation Index (ECVI)	16.04
ECVI for Saturated Model	27.08
ECVI for Independence Model	50.56
Independence Akaike Information Criterion (AIC)	1213.34
Model AIC	155.21
Saturated AIC	650.00
Independence Consistent Akaike Information Criterion (CAIC)	1268.81
Model CAIC	288.35
Saturated CAIC	1371.13
Normed Fit Index (NFI)	0.97
Non-Normed Fit Index (NNFI)	1.00
Comparative Fit Index (CFI)	1.00
Incremental Fit Index (IFI)	1.00
Root Mean Square Residual (RMR)	0.073
Standardized RMR	0.073
Goodness of Fit Index (GFI)	0.98
Adjusted Goodness of Fit Index (AGFI)	0.97
Parsimony Goodness of Fit Index (PGFI)	0.80

**Table 3:** Pearson’s correlation between selected variables and scores on Parental distress.

Variables	Parental Distress
Caregivers	
Gender	0.069
Age	0.082
Marital status	0.006
Educational status	−0.067
Employment status	0.100
Individual with disability	
Age	−0.072
Gender	−0.009
Number of services received	−0.072
Severity of Behavioral needs	0.224 **
Severity of Medical needs	0.168 **
Severity of the intellectual disability	0.176 **
Family capabilities	
Physical/Material Well-being	−0.170 **
Caregivers’ kinship	−0.150 **
Family interaction	−0.328 **
Parenting	−0.281 **
Family meaning	
Dysfunctional interaction	0.606 **
Difficult Child	0.580 **
Emotional Well-being	−0.357 **
Disability-Related Support	−0.224 **

** Significant with *p* < 0.01.

**Table 4:** Summary of hierarchical regression of Parental Distress.

Variables	Block 1					Block 2					Block 3				
	*B*	*SE*	*β*	*t*	*p*	*B*	*SE*	*β*	*t*	*p*	*B*	*SE*	*β*	*t*	*p*
Support needs															
Behavioral needs	0.71	0.19	0.17	3.68	<0.01	0.63	0.18	0.15	3.49	<0.01	−0.22	0.16	−0.05	−1.40	0.16
Medical needs	0.40	0.26	0.07	1.52	0.13	0.59	0.25	0.10	2.39	0.02	0.47	0.20	0.08	2.35	0.02
Severity of Intellectual disabilities	1.53	0.58	0.12	2.66	0.01	1.40	0.54	0.11	2.62	0.01	0.88	0.43	0.07	2.02	0.04
Family capabilities															
Kinship						−2.80	0.69	−0.16	−4.07	<0.01	−1.47	0.56	−0.09	−2.62	0.01
Physical/Material Wellbeing						0.25	0.56	0.02	0.45	0.65	0.03	0.51	0.00	0.05	0.96
Family Interaction						−2.99	0.69	−0.25	−4.33	<0.01	−1.68	0.58	−0.14	−2.91	<0.01
Parenting						−1.22	0.66	−0.11	−1.85	0.06	0.27	0.59	0.02	0.45	0.65
Family appraisal															
Dysfunctional interaction											0.39	0.05	0.35	7.57	<0.01
Difficult behaviors											0.29	0.05	0.28	5.79	<0.01
Emotional well-being											−1.69	0.45	−0.17	−3.81	<0.01
Disability-Related Support											0.63	0.51	0.06	1.24	0.22
*R*	0.253					0.453					0.700				
*R* ^2^	0.064 **					0.205 **					0.490 **				
Adj *R*^2^	0.058					0.194					0.479				
SE	9.83					9.10					7.31				
*F*(df_n_,df_d_)	F(3, 505) = 11.512 **					F(7, 501) = 18.451 **					F(11,497) = 43.384 **				

** *p* < 0.001. *B*—Regression coefficient; *SE*—Standard Error; *β*—Beta coefficient or standardized regression coefficient; *t*—T-values; *R*—Multiple Correlation Coefficient; *R*^2^—Coefficient of Determination; Adj *R*^2^—Adjusted Coefficient of Determination; SE—Standard Error of Prediction; *F*(dfn,dfd)—F-value and degrees of freedom.

## Supplementary Materials

The following are available online at https://www.mdpi.com/1660-4601/17/23/9007/s1, Table S1: Fully specified model.
