# Supplementary material for: Parental Stress and Family Quality of Life: Surveying Family Members of Persons with Intellectual Disabilities
Source: Int J Environ Res Public Health. 2020 Dec 3;17(23):9007. doi: 10.3390/ijerph17239007 (PMC7731363; doi:10.3390/ijerph17239007)
Supplement: Supplementary file 1 [file ijerph-17-09007-s001.pdf]

International Journal of
Environmental Research and Public Health

MDPI

Table S1. Fully specified model.

Variables	Block 1					Block 2					Block 3				
	B	*SE*	*β*	*t*	*p*	*B*	*SE*	*β*	*t*	*p*	*B*	*SE*	*β*	*t*	*p*
Individual characteristics															
Gender	−0.43	0.89	−0.02	−0.48	0.63	−0.82	0.82	−0.04	−1.00	0.32	−0.81	0.66	−0.04	−1.23	0.22
Age	−0.04	0.03	−0.06	−1.29	0.20	0.00	0.04	0.00	−0.07	0.95	0.00	0.03	0.00	−0.04	0.97
Severity of behavioral needs	0.68	0.20	0.16	3.48	<0.01	0.60	0.18	0.14	3.29	<0.01	−0.24	0.16	−0.06	−1.56	0.12
Severity of medical needs	0.41	0.26	0.07	1.54	0.12	0.58	0.24	0.10	2.39	0.02	0.47	0.20	0.08	2.37	0.02
Severity of Intellectual disabilities	1.60	0.58	0.13	2.76	0.01	1.84	0.55	0.14	3.36	<0.01	1.20	0.44	0.09	2.70	0.01
Family Capabilities															
Gender of caregiver						1.53	0.93	0.07	1.65	0.10	1.59	0.75	0.07	2.12	0.03
Age						−0.02	0.04	−0.02	−0.39	0.69	−0.02	0.04	−0.02	−0.53	0.60
Marital status						−0.56	0.93	−0.03	−0.61	0.55	0.28	0.75	0.01	0.38	0.71
Educational level						−0.61	0.37	−0.07	−1.63	0.10	−0.50	0.30	−0.06	−1.65	0.10
Employment status						2.04	0.93	0.10	2.19	0.03	1.38	0.76	0.07	1.83	0.07
Relationship						−2.45	0.95	−0.14	−2.59	0.01	−1.39	0.76	−0.08	−1.81	0.07
Physical/Material Wellbeing						0.48	0.56	0.04	0.86	0.39	0.28	0.51	0.02	0.55	0.58
Family Interaction						−2.99	0.69	−0.25	−4.32	<0.01	−1.69	0.58	−0.14	−2.93	<0.01
Parenting						−1.52	0.67	−0.14	−2.27	0.02	−0.09	0.61	−0.01	−0.14	0.89
Family appraisal															
Dysfunctional interaction											0.41	0.05	0.36	7.81	<0.01
Difficult behaviors											0.28	0.05	0.27	5.60	<0.01
Emotional well-being											−1.44	0.45	−0.14	−3.21	<0.01
Disability-Related Support											0.44	0.51	0.04	0.87	0.38
R	0.260					0.479					0.71				
R^2	0.068 **					0.230 **					0.507 **				
Adj R^2	0.058					0.208					0.489				
SE	9.8					9.02					7.25				
$F(df_n, df_d)$	F(3, 503) = 7.288 **					F(14, 494) = 10.513 **					F(18,490) = 27.979 **				

** $p < 0.001$.
